# Advances in prosthetics and orthotics

**DOI:** 10.1186/s12891-024-07246-y

**Published:** 2024-02-12

**Authors:** Gabriel Moisan, Christina Zong-Hao Ma

**Affiliations:** 1 Department of Human Kinetics, University du Québec à Trois-Rivières, 3351 Boulevard des Forges, Trois-Rivières, Québec G8Z 4M3 Canada; 2 Research Group on Neuromusculoskeletal Affections (GRAN), Trois-Rivières, G9A5H7 Canada; 3 Hong Kong Polytechnic University CN, Hung Hom, Hong Kong

## Abstract

Over the past years, the field of prosthetics and orthotics has seen incredible innovations that used to be perceived as science fiction. This editorial aims to shed light on such exciting developments, exploring how they are addressing the challenges faced by individuals with limb impairments and musculoskeletal conditions.

In recent years, there have been great improvements in how prosthetic limbs and wearable assistive/supportive devices like orthoses/braces are personalized for individuals. It’s not like the old days when everyone got the same type of prostheses or orthoses. Now, thanks to 3D printing and other digital techniques, these prosthetic and orthotic devices can be made to fit each person’s unique anatomy and needs. This makes them way more comfortable and works better for those who wear them. Patients who used to struggle with poorly fitted and uncomfortable prostheses or orthoses now experience a greater level of comfort and ease of movement. Furthermore, 3D printing will make these customized devices more accessible and cost-effective, breaking down financial barriers and ensuring that more people can benefit from these life-changing innovations [1].

Another groundbreaking development in the prosthetic field is the addition of neural, muscular and skeletal interfaces into prostheses. These neural (or brain-computer), muscular and skeletal interfaces allow patients to control their prostheses with their thoughts, which mimics the natural upper-limb and/or lower-limb movements [2, 3]. Some mind-controlled bionic arm with feedback technology even enabled the users to have the feeling of touching and grabbing an object [2]. These interfaces allow the users, for example, to pick up a cup of coffee or tie shoelaces mainly by using their minds. For individuals with an amputation and those with mobility impairments, this technology permits them to regain physical abilities, independence and autonomy.

Materials used in prosthetics and orthotics have also gotten much better. They are lightweight, durable, and comfortable. This supports fewer repairs and replacements, which used to be a hassle and expensive. Plus, modern prosthetics look more natural, which helps reduce the stigma that some people might feel about having a prosthetic limb which can have profound impacts on the psychological well-being and self-esteem of patients using these devices.

Patient-centered care has become a guiding principle in the field of Prosthetics and Orthotics, emphasizing the importance of education and rehabilitation programs. Making sure that patients are well-informed about the proper use and maintenance of their prosthetic or orthotic devices is essential for long-term success. Education programs help patients adapt to their devices, enabling them to optimize their mobility and independence. Rehabilitation is also crucial, helping individuals regain strength, coordination, and confidence as they adapt to their new reality.

The training, education, and nurturing of prosthetic and orthotic practitioners and professionals is another interesting topic and potential contribution for this collection. While the prosthetic and orthotic clinical practice is facing challenges from the current AI-empowered and digitalized era, the education and training of prosthetic and orthotic students, interns, and professionals shall also be advanced to cope with the need. There are always some opportunities lie in the difficulties and challenges. It would be exciting to see how efforts can be paid to promote the digital literacy in the prosthetic and orthotic educational field, how to balance the education of the conventional prostheses and orthoses and the state-of-the-art prosthetic and orthotic developments and solutions, etc.

Above and beyond the prosthetic and orthotic devices, some additional or adjunct innovative technologies can also be applied to validate and evaluate the functioning, effectiveness and efficiency of the prescribed devices on amputees and disabled persons, to facilitate the evidence-based practice and treatment. Examples include but are not limited to utilizing some wearable dynamic ultrasound imaging system to visualize amputee/patient’s muscle contraction pattern in different posture and during different activities, while using prostheses/orthoses with different designs, components, and/or alignments [4]; integrating the 3D printing technology with biomechanical stimulations (e.g., finite element model) to improve the prosthetics and orthotic designs to achieve a better prognosis and treatment outcome [5]; and identifying more parameters and characteristics to objectively evaluate and follow-up on the treatment effectiveness with AI and imaging technology [6], etc.

Even though the advancements in prosthetics and orthotics are significantly improved over the past few years, there is still much work to be done for the benefits of patients. Research in this field continues to push the boundaries, with ongoing studies on new technologies, materials, and treatment approaches. One domain of interest is biomechanics, which seeks to better understand the mechanics of human movement when wearing these devices [7]. This knowledge is crucial in designing prosthetic and orthotic devices that better restore mobility and enhance overall physical function. Deepening our understanding of the biomechanical effects of these devices will allow us to improve the quality of life in patients. The integration of robotics and sensors also holds immense promise. These technologies can enhance the adaptability and responsiveness of prosthetic and orthotic devices. Imagine a prosthetic leg that automatically adjusts to changes in terrain, providing a seamless and natural walking experience. Preventing prosthetic joint infections is another critical area of research as infections can lead to complications and discomfort for patients wearing prostheses. Developing effective prevention strategies is thus essential. Functional braces, compression garments, and splints also play a vital role in the field of orthotics. Research in these areas continues to refine and improve these devices, making them more effective and comfortable for patients.

The “Prosthetics and Orthotics” collection by BMC Musculoskeletal Disorders will show how much progress we have made in this field. Challenges that used to seem impossible are now being tackled with innovation and hard work. And the future looks bright! Prosthetics and Orthotics aren’t just about making people move better; they’re about giving people their dignity and independence back. So, all the experts and researchers out there, let’s keep pushing the boundaries and making life better for people with limb issues and musculoskeletal conditions. Together, we can create an even brighter future!

## Data Availability

No datasets were generated or analysed during the current study.
